# Durable Response Following the Discontinuation of Immune Checkpoint Inhibitor Therapy in Advanced Non-small Cell Lung Cancer

**DOI:** 10.7759/cureus.83610

**Published:** 2025-05-06

**Authors:** Taiyo Nakamura, Yohei Kawaguchi, Jiyunichirou Oosawa, Kentaro Imai, Takuya Aoki, Naohiro Kajiwara, Norihiko Ikeda

**Affiliations:** 1 Department of Thoracic Surgery, Tokyo Medical University Hachioji Medical Center, Hachioji, JPN; 2 Department of Surgery, Tokyo Medical University, Tokyo, JPN; 3 Department of Clinical Oncology, Tokyo Medical University Hachioji Medical Center, Hachioji, JPN

**Keywords:** best supportive care, immune checkpoint inhibitor, immune-related adverse events, non-small cell lung cancer, subsequent chemotherapy, the optimal duration of ici therapy

## Abstract

Background

The optimal duration of immune checkpoint inhibitor (ICI) therapy for maximum benefits remains unclear. Recently, the long-term follow-up data from clinical trials suggest the existence of a durable response (DR) that maintains the therapeutic effect even after ICI discontinuation. The study aimed to explore how the characteristics of ICI therapy influence the effectiveness of subsequent treatments in patients with advanced non-small-cell lung cancer (NSCLC).

Methods

The medical records of 134 patients with NSCLC who received ICIs before December 31, 2022, were retrospectively reviewed. We evaluated the impact of pretreatment ICIs on survival after completion of ICI administration.

Results

Among the 116 included patients, long ICI use (≥180 days) was the only independent prognostic factor for post-ICI overall survival (OS) in the multivariate analysis (HR: 0.382, 95% CI: 0.206-0.708, *p*=0.002). Patients who received ICIs for < 180 days showed significantly improved survival with subsequent chemotherapy (SC) compared to those who received only best supportive care (BSC) (p<0.001). However, among patients treated with ICIs for ≥ 180 days, no significant difference in OS or post-ICI OS was observed between the SC and BSC groups (p=0.188). In patients who discontinued ICIs due to PD, the impact of ICI treatment duration on survival outcomes differed. Among those with short ICI use, the SC group showed significantly better post-ICI OS compared to the BSC group (p=0.007). However, in patients with long ICI use, there was no significant difference in post-ICI OS between the SC and BSC groups (p=0.913). Regarding OS, no statistically significant differences were observed between the SC and BSC groups, regardless of ICI treatment duration. The 2-year OS was 47.6% in the SC group and 46.0% in the BSC group among patients with short ICI use (p=0.549), and 93.3% vs. 66.7% among those with long ICI use (p=0.136). Similarly, in patients who discontinued ICIs without PD, survival outcomes varied depending on ICI duration. Among those with short ICI use, the BSC group had a 2-year post-ICI OS of 21.1%, which was lower than that of the SC group (50.0%; p=0.08). The 2-year OS was also significantly higher in the SC group (64.3%) compared to the BSC group (31.6%; p=0.008). In contrast, no significant differences were observed in post-ICI OS (p=0.104) or OS (64.3% vs. 31.6%; p=0.104) between the SC and BSC groups among patients with long ICI use.

Conclusion

The achievement of a DR through prolonged ICI use may reduce the need for immediate subsequent chemotherapy in selected patients.

## Introduction

Immune checkpoint inhibitors (ICIs) have notably changed the treatment approach for advanced non-small-cell lung cancer (NSCLC), providing patients with the potential for prolonged survival. Several clinical trials have set a standard ICI treatment duration based on initial efficacy and safety assumptions. However, the optimal duration of ICI therapy for maximum benefit remains unclear [1-4].

Recent long-term follow-up data from clinical trials revealed patient outcomes beyond the defined treatment period. In the KEYNOTE-010 trial, 79 of 690 patients completed 2 years of pembrolizumab therapy, and their 2-year progression-free survival after completion was 57.7% [1,5]. Similarly, in the KEYNOTE-024 trial, 39 patients completed 2 years of pembrolizumab therapy, with the majority (82%) still alive at 5 years [6]. These findings suggest the existence of a "durable response (DR)" that maintains the therapeutic effect even after ICI discontinuation. Several hypotheses have been proposed to explain this phenomenon, such as the sustained expression of PD-1 receptors on circulating T cells for several months and the development of adaptive immune responses through memory T cells in the tumor microenvironment [7].

Studies on various cancer types have reported that patients who discontinue ICIs without disease progression show long-lasting responses regardless of the reason for discontinuation [8,9]. Real-world data from Korea on ICI use in NSCLC have demonstrated the potential for achieving DR and promoting long-term survival even when therapy is discontinued after six months [10]. When treatment is stopped for various reasons, including disease progression, patients typically undergo subsequent chemotherapy. We hypothesized that the DR associated with ICIs may also affect the efficacy of post-ICI chemotherapy.

Study objectives

This study aimed to examine whether prolonged ICI use independently contributes to a DR, and whether subsequent chemotherapy after ICI discontinuation improves survival outcomes in patients with advanced NSCLC.

## Materials and methods

Study design and settings

This single-center observational study focused on the long-term outcomes of patients after ICI therapy.

Patient selection

Overall, 134 consecutive patients with NSCLC, treated with ICIs between January 2016 and December 2022 at the Tokyo Medical University Hachioji Medical Center, were initially included in this study. Patients who received ICIs as postoperative adjuvant chemotherapy and those who received durvalumab after chemoradiotherapy were excluded. All patients were diagnosed according to the 8th edition of the TNM Classification for Lung and Pleural Tumors of the Union for International Cancer Control [11]. Eighteen patients who were still receiving ICIs at the time of the study were excluded from the final selection. In total, 116 patients were included in this study. Information on the use of maintenance therapies, including pemetrexed and bevacizumab, was reviewed. No patients in this cohort continued pemtrexed or bevacizumab maintenance therapy after ICI discontinuation. Patients who were still receiving maintenance therapy concurrently with ICIs at the time of data cutoff were considered to have ongoing treatment and were therefore excluded from the analysis cohort.

Data collection

**Figure 1:**
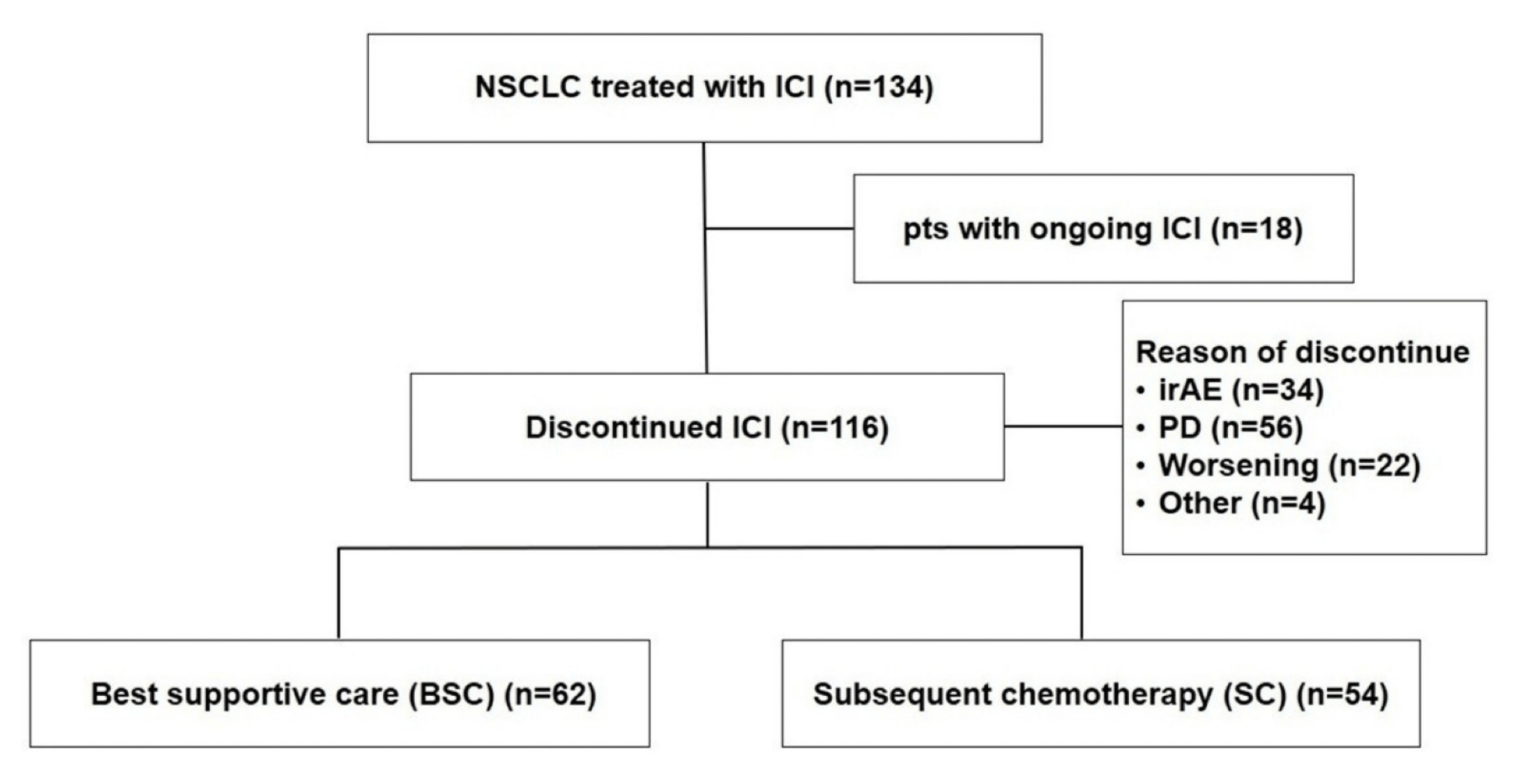
Flow chart of this study NSCLC, non-small cell lung cancer; ICI, immune checkpoint inhibitor; irAE, immune-related adverse events; PD, progressive disease

Detailed clinicopathological data of the patients, including demographic details, disease characteristics, treatment regimens, and follow-up data, were systematically collected from the medical records. Clinical information was extracted by Dr. Nakamura and Dr. Kawaguchi. As the data were directly retrieved from the electronic records, there were no missing values that affected the analyses. Complete blood counts and biochemical data were also retrieved. The neutrophil-to-lymphocyte ratio (NLR), which has been reported to affect ICI efficacy, was calculated using these variables [12,13]. NLR was computed as the absolute neutrophil count divided by the absolute lymphocyte count. The cutoff value for testing the association of NLR with ICI efficacy was set at 5 based on previous reports [14]. Patients were categorized into two groups: those who received best supportive care (BSC) after discontinuing ICIs (BSC group) and those who received subsequent chemotherapy after discontinuing ICI (SC group; Figure 1).

Pathological evaluation

All collected specimens were fixed in 10% formalin and embedded in paraffin. Representative sections were stained with hematoxylin and eosin and reviewed by experienced pathologists to confirm the inclusion of carcinoma cells. The quality of specimens was adequate for analysis, and inter-observer variability was minimal. PD-L1 positivity in histological specimens was defined as ≥1% membranous staining through immunohistochemistry using the 22C3 pharmDx test (Dako North America, Carpinteria, CA, USA) [15,16].

Outcome measurement

In the present study, we defined two outcomes of prognostic analyses of patients after ICI use: overall survival (OS) and OS after discontinuation of ICIs (post-ICI OS). OS was defined as the time from the date of initial administration of first line chemotherapy to the date of follow-up completion or death, whereas post-ICI OS was defined as the time from the date of ICI discontinuation to the date of follow-up completion or death.

Statistical analysis

Categorical variables were compared using the chi-square test and analysis of variance (ANOVA). Kaplan-Meier curves for OS and post-ICI OS were analyzed, and differences in survival rates were determined using the log-rank test. The Cox proportional hazards test was used to calculate hazard ratios (HRs) and their 95% confidence intervals (CIs) to identify independent predictors of OS and post-ICI OS. Significant variables (p < 0.05) in the univariate analysis were included in the multivariate Cox regression model using the enter method. All significant variables identified in the univariate analysis were included in the multivariate model using the forced-entry method, allowing for appropriate adjustment of potential confounders. Statistical significance was set at p < 0.05. Statistical analyses were conducted using the SPSS statistical software package (version 29.0; IBM Corp., Armonk, NY, US).

## Results

Patients

The patient characteristics are shown in Table 1. Of 116 patients, 89 (76.7%) were men, 78 (67.2%) were aged < 75 years, and 33 (28.4%) had an NLR of < 5. Histological types included 62 patients (53.4%) with adenocarcinoma, 32 patients (27.6%) with squamous cell carcinoma, and 22 patients (19.0%) classified as others (20 NSCLC and 2 large cell neuroendocrine carcinoma). PD-L1 status was negative (< 1%) in 51 patients (44.0%) and strongly positive (≥ 50%) in 32 patients (27.6%). A total of 86 patients (74.1%) received ICIs as first-line treatment. Pembrolizumab was the most used ICI (72 patients, 62.1%). The duration of ICI administration was categorized based on a cutoff of 180 days, referring to prior real-world data suggesting that discontinuation after 6 months could still achieve DR and promote long-term survival [10]. Based on this evidence, we hypothesized that patients who received ICI for at least 180 days would experience durable responses and might not derive additional survival benefits from subsequent chemotherapy. Thirty-five patients (30.2%) received ICIs for more than 180 days. Reasons for discontinuing ICIs included progressive disease (PD) in 56 patients (48.3%) and immune-related adverse events (irAEs) in 34 patients (29.3%). Other reasons for ICI discontinuation included three cases of other deaths and one case of worsening of another disease. There was a significant difference between the BSC and SC groups for NLR (p=0.002), treatment line (p=0.032), and ICI using days (p=0.021).

**Table 1:** Patient characteristics BSC; best supportive care, NLR; neutrophil to lymphocyte ratio, PD-L1; programmed death-ligand 1, EGFR; epidermal growth factor receptor, ALK; anaplastic lymphoma kinase, ICI; immune checkpoint inhibitor, CR, complete response, PR; partial response, SD; stable disease, PD; progression disease, NE; not evaluable, irAE; immune-related adverse events, PS; performance status

Variable	All patients (n=116) (%)	BSC (n=62) (%)	SC (n=54) (%)	P value	chi-square value/F-value
Sex				0.051	3.809
Male	89 (76.7)	52 (83.9)	37 (68.5)		
Female	27 (23.3)	10 (16.1)	17 (31.5)		
Age: median (range) (years)	73 (53-85)			0.143	2.141
<75	78 (67.2)	38 (61.3)	40 (74.1)		
≧75	38 (32.8)	24 (38.7)	14 (25.9)		
Smoking index: median (range)	700 (0-2850)			0.272	1.205
≦200	35 (30.2)	16 (25.8)	19 (35.2)		
>200	81 (69.8)	46 (74.2)	35 (64.8)		
NLR: median (range)	3.4 (0.4-22.3)			0.002	9.226
≤5	33 (28.4)	25 (40.3)	8 (14.8)		
>5	83 (71.6)	37 (59.7)	46 (85.2)		
Histological type				0.489	0.481
Adenocarcinoma	62 (53.4)	38 (61.3)	24 (44.4)		
Squamous cell carcinoma	32 (27.6)	14 (22.6)	18 (33.3)		
Others	22 (19.0)	10 (16.1)	12 (22.2)		
PD-L1 status, No. (%)				0.798	0.066
≥50	32 (27.6)	18 (29.0)	14 (25.9)		
1< ≤49	33 (28.4)	17 (27.4)	16 (29.6)		
<1	51 (44.0)	27 (43.5)	24 (44.4)		
EGFR mutation status				0.051	3.824
Mutant	9 (7.8)	2 (3.2)	7 (13.0)		
Wild type/unknown	107 (92.2)	60 (96.8)	47 (87.0)		
ALK translocation present				0.282	1.158
Presence	1 (0.9)	0 (0.0)	1 (1.9)		
Absence/unknown	115 (99.1)	62 (100.0)	53 (98.1)		
Treatment line				0.032	4.580
1	86 (74.1)	51 (82.3)	35 (64.8)		
≥2	30 (25.9)	11 (17.7)	19 (35.2)		
Treatment				0.514	0.426
Immunotherapy	51 (44.0)	29 (46.8)	22 (40.7)		
chemoimmunotherapy	65 (56.0)	33 (53.2)	32 (59.3)		
Type of ICI				0.083	3.067
Nivolumab±Ipilimumab	24 (20.7)	11 (17.7)	13 (24.1)		
Pembrolizumab	72 (62.1)	43 (69.4)	29 (53.7)		
Atezolizumab	20 (17.2)	8 (12.9)	12 (22.2)		
ICI using date: median (range) (days)	88 (1-1544)			0.021	5.356
<180	81 (69.8)	49 (79.0)	32 (59.3)		
≥180	35 (30.2)	13 (21.0)	22 (40.7)		
Best overall response				0.058	3.661
CR	1 (0.9)	1 (1.6)	0 (0.0)		
PR	57 (49.1)	26 (41.9)	31 (57.4)		
SD	18 (15.5)	9 (14.5)	9 (16.7)		
PD	33 (28.4)	20 (32.3)	13 (24.1)		
NE	7 (6.0)	6 (9.7)	1 (1.9)		
Reason of discontinuation				0.852	0.035
irAE	34 (29.3)	26 (41.9)	8 (14.8)		
PD	56 (48.3)	15 (24.2)	41 (75.9)		
Worsening PS	22 (19.0)	18 (29.0)	4 (7.4)		
Others	4 (3.4)	3 (4.8)	1 (1.9)		

Survival outcomes

**Figure 2:**
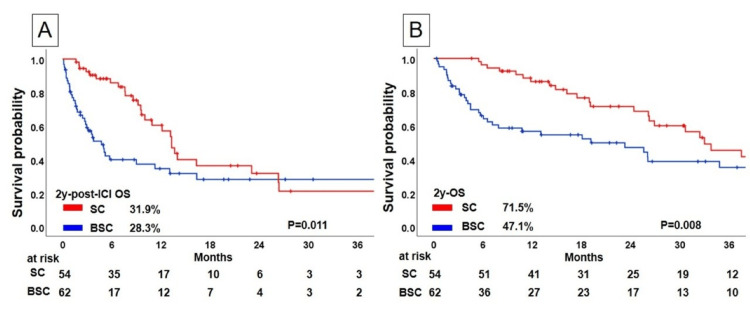
Kaplan-Meier analysis of survival profiles of NSCLC patients with immunochemotherapy; Comparison of (A) post-ICI overall survival and (B) overall survival between BS and SC NSCLC, non-small cell lung cancer; ICI, immune checkpoint inhibitor; BS, best supportive care; SC, subsequent chemotherapy after discontinuing ICI

The Kaplan-Meier curves for post-ICI OS and OS according to subsequent treatment after ICI therapy are illustrated in Figure 2. The 2-year post-ICI OS rate in the SC group was 31.9%, and that of the BSC group was 28.3%. The SC group showed a significantly favorable post-ICI OS compared with that of the BSC group (p=0.011) (Figure 2A). The 2-year OS rate in the SC group was 71.5%, and that of the BSC group was 47.1%. The SC group showed a significantly favorable OS compared to that of the BSC group (p=0.008; Figure 2B).

Univariate and multivariate analyses were conducted to assess post-ICI OS and OS (Tables 2, 3). Long ICI use (≥180 days) was the only prognostic factor for post-ICI OS (HR: 0.382, 95% CI: 0.206-0.708, p=0.002) in multivariate analysis (Table 2). In the multivariate analysis of OS, long ICI use was also a prognostic factor (HR, 0.331; 95% CI: 0.65-0.603, p<0.001; Table 3).

**Table 2:** Univariate and multivariate analysis for post ICI-OS OS; overall survival, SI; smoking index, ICI; immune checkpoint inhibitor, NLR; neutrophil to lymphocyte ratio, TPS; tumor proportion score, Chemo; chemotherapy

		Univariate analysis			Multivariate analysis		
Variables	Risk factor	HR	95%CI	p-value	HR	95%CI	p-value
Sex	Female	0.828	0.461-1.487	0.528	-	-	-
Age	≤75	0.985	0.947-1.024	0.441	-	-	-
SI	≥200	0.786	0.439-1.407	0.418	-	-	-
Histology	Non-adenocarcinoma	0.819	0.498-1.348	0.432	-	-	-
Treatment	Chemo+ICI	0.954	0.577-1.576	0.853	-	-	-
Line of ICI	1^st^ line	0.738	0.443-1.231	0.245	-	-	-
NLR	≤5	0.556	0.325-0.951	0.032	0.700	0.395-1.240	0.222
ICI using days	≥180 days	0.367	0.198-0.678	0.001	0.382	0.206-0.708	0.002
TPS	≥50%	0.702	0.380-1.297	0.259	-	-	-
Post treatment with ICI	Yes	0.512	0.0.311-0.844	0.009	0.588	0.346-0.999	0.050

**Table 3:** Univariate and multivariate analysis for OS OS; overall survival, SI; smoking index, ICI; immune checkpoint inhibitor, NLR; neutrophil to lymphocyte Ratio, TPS; tumor proportion score, Chemo; chemotherapy

		Univariate analysis			Multivariate analysis		
Variables	Risk factor	HR	95%CI	p-value	HR	95%CI	p-value
Sex	Male	0.548	0.295-1.019	0.057	-	-	-
Age	≦75	0.879	0.518-1.491	0.633	-	-	-
SI	<200	0.534	0.300-0.951	0.033	0.751	0.412-1.369	0.350
Histology	Non-adenocarcinoma	0.742	0.449-1.227	0.245	-	-	-
Treatment	ICI	0.841	0.495-1.393	0.481	-	-	-
Line of ICI	1^st^ line	0.584	0.336-1.014	0.056	-	-	-
NLR	5>	0.687	0.403-1.174	0.170	-	-	-
ICI using days	≥ 180 days	0.275	0.146-0.518	<0.001	0.331	0.165-0.603	<0.001
TPS	≥ 50%	0.793	0.428-1.470	0.642	-	-	-
Post treatment after ICI	Yes	0.517	0.310-0.860	0.011	0.662	0.389-1.128	0.129

To further understand the prognostic factors after ICI treatment, we classified SC and BSC into two groups according to the duration of ICI use (SC patients with long ICI use: L-SC; SC patients with short (<180 days) ICI use: S-SC; BSC patients with long ICI use: L-BSC; and BSC patients with short ICI use: S-BSC).

Survival outcomes according to the treatment duration of ICIs

**Figure 3:**
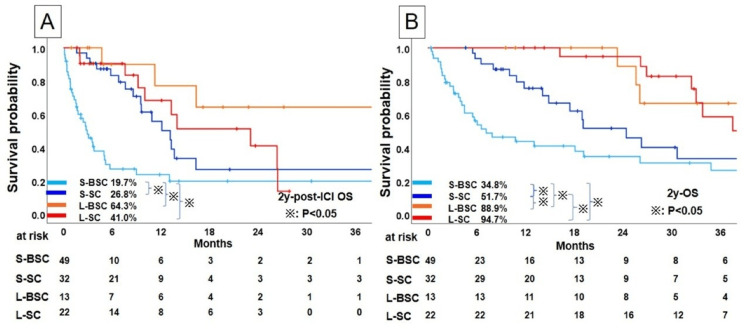
Kaplan-Meier analysis of the survival profiles of NSCLC patients treated with immunochemotherapy; comparison of S-BSC, S-SC, L-BSC, and L-SC in (A) post-ICI overall survival and (B) overall survival S; shorter, L; longer; BSC; best supportive care, SC; chemotherapy after discontinuation of immune checkpoint inhibitor

Figure 3A shows the post-ICI OS according to treatment duration and chemotherapy after ICI therapy. The S-SC group showed a 2-year post-ICI OS rate of 26.8% and a favorable prognosis as compared with that of the S-BSC group (2-year post-ICI OS: 19.7%; p<0.001). In contrast, the patients treated with ICIs for long durations showed no statistical difference between the L-SC (2-year post-ICI OS: 41.0%) and L-BSC groups (2-year post-ICI OS: 64.3%; p=0.188). In the OS analysis, although the S-SC group (2-year OS: 51.7%) showed favorable OS compared to that of the S-BSC group (2-year OS: 34.8%), no statistical difference was observed between the L-SC (2-year OS: 94.7%) and L-BSC groups (2-year OS: 88.9%; p=0.615) (Figure 3B). Additionally, because the prognostic impact of ICI duration may differ depending on whether discontinuation occurred due to PD, we stratified the subsequent analyses by PD versus non-PD cases to more clearly evaluate the presence of a DR.

Survival outcomes according to the reason for discontinued ICIs

**Figure 4:**
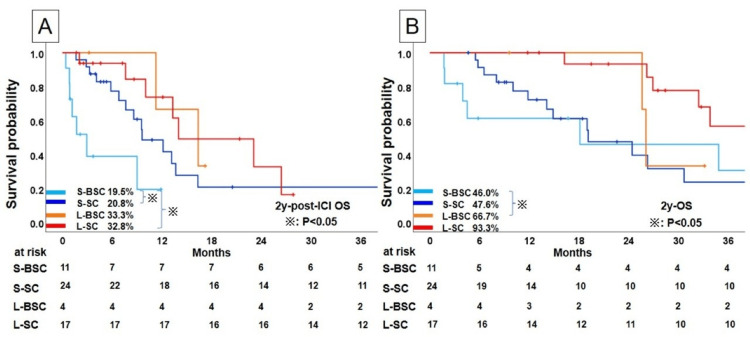
Kaplan-Meier analysis of survival profiles of NSCLC patients who discontinued immunochemotherapy for PD; (A) post-ICI overall survival and (B) overall survival NSCLC, non-small cell lung cancer; PD, progressive disease; ICI, immune checkpoint inhibitor; BSC, best supportive care; SC, chemotherapy after discontinuation of ICI; S, shorter; L, longer

Figure 4 shows the post-ICI OS and OS rates according to the treatment duration of ICIs in patients who discontinued ICIs for PD.

In patients with short ICI use, there was a significant difference between the S-SC and S-BSC groups in post-ICI OS (p=0.007), whereas in patients with long ICI use, there was no difference in post-ICI OS between the L-SC and L-BSC groups (p =0.913; Figure 4A).

In OS analysis, no statistical differences were observed between the SC and BSC groups in both patients with short ICI use (2-year OS; S-SC: 47.6%, S-BSC: 46.0%, p=0.549) or patients who had long ICI use (2-year OS; L-SC: 93.3%, L-BSC: 66.7%, p= 0.136); Figure 4B).

**Figure 5:**
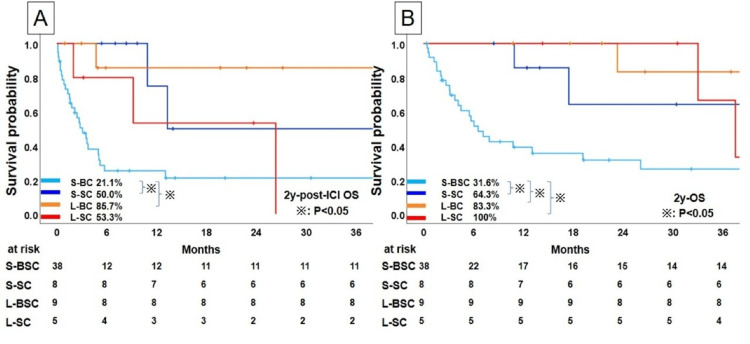
Kaplan-Meier analysis of survival profiles of NSCLC patients who discontinued immunochemotherapy for other than PD. (A) Post-ICI overall survival and (B) overall survival. NSCLC, non-small cell lung cancer; PD, Progressive Disease; ICI, immune checkpoint inhibitor; BSC, best supportive care; SC, chemotherapy after discontinuation of ICI; S, shorter; L, longer

The post-ICI OS and OS rates according to the treatment duration of ICIs in patients who discontinued ICIs without PD are shown in Figure 5.

In patients with short ICI use, the S-BSC group showed 21.1% with a 2-year post-ICI OS and a significantly poorer prognosis compared with those in the S-SC group (2-year post-ICI OS: 50.0%; p=0.08); whereas the patients with long ICI use showed no significant difference between the L-SC and L-BSC groups (p=0.104) (Figure 5A).

In the OS analysis of patients who discontinued ICIs without PD, as in previous analyses, there was a significant difference between SC and BSC in patients with short ICI use (2-year OS: S-SC, 64.3%; S-BSC, 31.6%; p =0.008) but not in patients who had long ICI use (2-year OS: S-SC, 64.3%; S-BSC, 31.6%; p=0.104; Figure 5B).

## Discussion

The present study investigated the effect of ICI treatment duration and subsequent chemotherapy on the prognosis of patients with advanced NSCLC after ICI discontinuation. Our findings revealed that the duration of pretreatment ICI use considerably influenced prognosis after ICI completion. Notably, in the group where previous ICIs were administered for more than 180 days, the presence or absence of chemotherapy after ICI discontinuation did not affect the prognosis, regardless of the reason for ICI discontinuation.

These results suggest that DR was achieved in patients who received ICIs for a sufficient period of time. This observation aligns with previous studies that reported long-lasting responses in patients who discontinued ICIs without disease progression, irrespective of the reason for discontinuation [17]. The sustained therapeutic effect after ICI discontinuation may be due to the development of adaptive immune responses through memory T cells in the tumor microenvironment and the persistent expression of PD-1 receptors on circulating T cells for several months [18]. Several prospective studies have investigated the optimal duration of ICI administration. A prospective study was conducted in previously treated patients with NSCLC who were still receiving nivolumab monotherapy after one year and were randomly assigned to continue or discontinue nivolumab until disease progression, unacceptable toxicity, or discontinuation of nivolumab (CheckMate 153) occurred [19]. The results of this trial showed that discontinuation of ICI therapy in patients with NSCLC was associated with poor progression-free survival and OS. Another prospective study was conducted to compare the efficacy and incidence of irAEs by randomizing patients who had a good response to PD-1 pathway inhibitors for at least 12 months into a discontinuation group and a continuation group [20]. Although these trials set the duration of ICI treatment at one year, our validation suggests that even six months may have been a sufficient treatment period to obtain DR. In our cohort, 29.3% of patients discontinued ICIs due to irAEs, which is a higher proportion compared to previous clinical trials. This may reflect real-world patient characteristics, such as a higher prevalence of elderly patients and those with complications, as well as differences in physician decision-making thresholds regarding ICI discontinuation. Moreover, in daily clinical practice, patients with severe irAEs often transitioned to best supportive care, contributing to the elevated proportion in the BSC group.

Interestingly, our study found that continuous chemotherapy did not necessarily prolong the prognosis in patients who had received ICI for an adequate duration. These findings question the conventional notion that subsequent chemotherapy is beneficial after ICI discontinuation. It is possible that DR obtained from prolonged ICI use may have a more notable impact on long-term survival than the additional benefit provided by subsequent chemotherapy. Our findings have important implications for future treatment strategies. In patients who have received ICI for a sufficient period, a chemotherapy-free interval may be considered a potential treatment option. This strategy could reduce the toxicity and financial burden associated with continuous chemotherapy, while maintaining the benefits of the achieved DR.

Our study has several limitations. Several limitations and biases in this study should be considered when interpreting the results. First, as a retrospective, single-institution study, potential biases, such as selection bias, information bias, and unmeasured confounding factors, could not be fully excluded. Second, the sample size was relatively small, particularly in subgroup analyses, which may have limited the statistical power and reduced the reliability of the conclusions. Third, in our cohort, we identified a treatment duration of 180 days as the threshold for achieving a durable response. However, whether this cut-off can be applied to other patient populations remains uncertain and requires further investigation. Importantly, the BSC group included patients who discontinued ICI early due to irAEs and subsequently underwent active surveillance without disease progression. Several studies have reported that patients who discontinued ICIs early because of irAEs have a favorable prognosis [9,21-24]. This heterogeneity within the BSC group may have influenced survival outcomes and limited the interpretation of the group comparisons. Future large-scale prospective studies with more detailed subgroup stratification are warranted to validate our findings and refine treatment strategies.

## Conclusions

This study investigated the effect of ICI treatment duration and subsequent chemotherapy on the prognosis of patients with advanced NSCLC after ICI discontinuation. Our findings demonstrated that the duration of pretreatment ICI use significantly influenced post-ICI prognosis. Notably, in patients who received ICIs for 180 days or more, the presence or absence of chemotherapy after discontinuation did not impact survival, regardless of the reason for ICI termination. These results suggest that DR was achieved in patients treated with ICIs for a sufficient period, aligning with previous reports showing DRs even after treatment discontinuation without disease progression. Our findings indicate that a treatment period of approximately six months may be sufficient to induce DR in some patients. Interestingly, continued chemotherapy did not necessarily improve prognosis in patients who had already received ICIs for an adequate duration. This questions the conventional approach of routinely administering chemotherapy after ICI discontinuation. DR achieved through prolonged ICI therapy may have a greater influence on long-term survival than the incremental benefit of subsequent chemotherapy. These insights have meaningful implications for future treatment strategies. In selected patients with sufficient ICI exposure, a chemotherapy-free interval may be considered a feasible approach, potentially minimizing treatment-related toxicity and financial burden while maintaining clinical benefit.
